# The Role of Mentalizing and Alexithymia in Burnout Among Physicians

**DOI:** 10.7759/cureus.113320

**Published:** 2026-07-24

**Authors:** Aleksandar D Radlovic, Andjela Gogic, Nebojsa Zdravkovic, Branimir Radmanovic

**Affiliations:** 1 Faculty of Medical Sciences, University of Kragujevac, Kragujevac, SRB; 2 Department of General Medicine, Primary Health Care Center Nikšić, Nikšić, MNE; 3 Faculty of Medical Sciences, Department of Medical Statistics and Informatics, University of Kragujevac, Kragujevac, SRB; 4 Psychiatry Clinic, University Clinical Center Kragujevac, Kragujevac, SRB

**Keywords:** alexithymia, burnout, depersonalization, emotional exhaustion, mentalization, montenegro, physicians, reflective functioning, reflective functioning questionnaire (rfq-8), toronto alexithymia scale (tas-20)

## Abstract

Background

Burnout among physicians is a major global public health problem with significant implications for both healthcare professionals and patient care. This study aimed to examine the associations between sociodemographic characteristics, alexithymia, mentalizing capacity, and different dimensions of burnout among physicians in Montenegro, as well as to assess their independent contributions.

Methods

A cross-sectional study was conducted among 235 physicians employed in primary, secondary, and tertiary healthcare settings. Burnout was assessed using the Maslach Burnout Inventory, alexithymia using the Toronto Alexithymia Scale (TAS-20), and mentalizing capacity using the Reflective Functioning Questionnaire (RFQ-8). Nonparametric analyses, correlation analyses, and hierarchical linear regression were applied.

Results

The regression model showed that emotional exhaustion was positively associated with hypomentalization (β = 0.17, p < 0.05), while hypermentalization (β = -0.47, p < 0.001) and alexithymia (β = -0.20, p < 0.01) were negatively associated, with the model explaining 40.0% of the variance (R² = 0.400). For depersonalization, hypomentalization (β = 0.19, p < 0.05) and hypermentalization (β = -0.26, p < 0.001) were significant predictors, with the model explaining 20.6% of the variance (R² = 0.206). Alexithymia was not statistically significant. No psychological variable was significantly associated with personal accomplishment.

Conclusion

Mentalizing capacity appears to be an important factor associated with burnout among physicians, while the role of alexithymia appears limited and context-dependent. These findings may have implications for the development of interventions aimed at supporting physicians’ mental health in high-stress healthcare environments.

## Introduction

Healthcare workers, particularly physicians, are continuously exposed to a substantial psychological burden due to the specific demands of their profession. These demands include high levels of responsibility, time pressure, and decision-making under conditions of uncertainty. Physicians are often emotionally involved in the care of seriously ill patients. They are exposed to organizational problems such as shift work, administrative burden, and very limited resources. Long-term exposure to such working conditions can lead to chronic exhaustion and the development of burnout syndrome [1, 2].

Burnout at work is conceptualized as a prolonged response to chronic emotional and interpersonal stressors in the workplace [3]. According to the model proposed by Maslach, burnout encompasses three basic dimensions: emotional exhaustion, depersonalization, characterized by a cynical and distant attitude towards patients and work; and reduced personal achievement [4]. Emotional exhaustion reflects a state of excessive strain and depletion of emotional resources [4, 5]. Depersonalization represents emotional distancing and indifference towards patients, while reduced personal achievement involves negative self-evaluation and reduced professional efficacy [4, 5].

Burnout among physicians is a significant global public health problem. In the United States, during the COVID-19 pandemic, the prevalence of burnout among physicians increased from 38.2% in 2020 to 62.8% in 2021, significantly higher than that observed in many other professions [6]. Key factors contributing to burnout include workload, work-life balance, organizational support, and sociodemographic characteristics such as age, gender, and years of professional experience [6]. Given that burnout has important consequences for both physician mental health and the quality and safety of patient care [7, 8], increasing attention has been directed at examining individual psychological factors that may influence susceptibility to burnout.

Alexithymia, also known as "emotional blindness" or emotional illiteracy, is a disorder characterized by difficulty recognizing emotions and a tendency toward external focus rather than introspective thinking [9-11]. Severe alexithymia may be associated with increased anxiety, depression, and impaired emotional regulation, which may predispose individuals to burnout at work [12]. Large population-based studies, such as the Finnish Health Study of 2000, have shown that alexithymia and its subdimensions are significantly associated with burnout, independent of depression and sociodemographic variables [13].

Mentalizing capacity, also referred to as reflective functioning, denotes the ability to understand one’s own and others’ mental states, including emotions, intentions, and desires [14]. Two maladaptive forms of mentalizing capacity can be distinguished: hypermentalizing and hypomentalizing. Hypermentalizing involves excessive and overconfident attribution of mental states to others, whereas hypomentalizing reflects a reduced capacity to recognize and interpret mental states, leading to uncertainty in understanding others’ behavior [14]. However, the interpretation of certainty about mental states, particularly when assessed using the Reflective Functioning Questionnaire (RFQ-8), remains a matter of ongoing debate. In non-clinical populations, higher RFQ-C scores may reflect greater certainty in understanding mental states rather than pathological hypermentalizing. Since alexithymia involves deficits in emotional awareness and expression, it may impair mentalizing capacity and reduce the ability to process emotional information adaptively [15, 16]. In contrast, adequate mentalizing capacity supports emotional regulation, professional functioning, and interpersonal relationships, thereby acting as a protective factor against burnout [15, 16].

Within the Job Demands-Resources (JD-R) framework, burnout is understood as a consequence of a chronic imbalance between job demands and available psychological resources. Previous studies on burnout syndrome have more frequently examined age as a sociodemographic factor. Therefore, following the approach used in previous research, the authors decided to include age as a variable in the present study. From this perspective, individual factors such as emotional processing and interpersonal functioning may represent important personal resources that influence adaptation to occupational stress. Accordingly, burnout can also be conceptualized as being associated with impairments in emotional processing and interpersonal functioning. The JD-R model emphasizes that burnout develops as a result of the imbalance between job demands and available job resources. According to this framework, high occupational demands contribute to emotional exhaustion, whereas adequate job resources may reduce the risk of burnout and promote employee well-being [17]. 

Alexithymia reflects deficits in emotional awareness and expression, which may hinder the ability to accurately identify and regulate internal states [18, 19]. These deficits may, in turn, be associated with reduced mentalizing capacity, particularly in the form of hypomentalizing, characterized by difficulties in understanding one’s own and others’ mental states [20]. Impaired mentalizing capacity may be linked to maladaptive responses to occupational stress, which may be associated with higher levels of emotional exhaustion and depersonalization [21]. In contrast, more adaptive mentalizing capacity may be related to more effective emotional regulation, resilience, and interpersonal functioning. However, this should be interpreted with caution, as certain forms of mentalizing capacity, such as hypermentalizing, are generally conceptualized as biased or maladaptive [20, 21].

Taken together, alexithymia and mentalizing capacity represent key psychological constructs involved in emotional processing and adaptation to stress. Although both have been individually associated with burnout, their combined effects and interrelationship remain insufficiently explored, particularly among physicians exposed to high occupational demands. A clearer understanding of these mechanisms may support the development of targeted interventions aimed at improving emotional functioning and reducing burnout in clinical settings. The relevance of examining these relationships is particularly pronounced in the context of the Montenegrin healthcare system, which is characterized by limited resources, chronic workforce shortages, high administrative burden, and frequent shift work. These factors represent substantial occupational stressors and may contribute to an increased risk of burnout among physicians [22].

The present study aimed to examine the relationships among sociodemographic characteristics, alexithymia, mentalizing capacity, and different dimensions of burnout in a sample of physicians in Montenegro. To the best of our knowledge, no previous studies have investigated this issue among physicians in Montenegro. The study sought to assess the independent contributions of alexithymia and mentalizing capacity to burnout after controlling for relevant sociodemographic factors.

Based on the theoretical framework and previous empirical findings, it was hypothesized that higher levels of hypomentalizing would be positively associated with emotional exhaustion and depersonalization, whereas higher levels of hypermentalizing would be negatively associated with these burnout dimensions. Alexithymia was expected to be associated with burnout, particularly emotional exhaustion, and to be positively associated with hypomentalizing while being negatively associated with hypermentalizing. Furthermore, it was hypothesized that mentalizing capacity dimensions and alexithymia would significantly contribute to the prediction of burnout dimensions after controlling for sociodemographic and work-related variables.

## Materials and methods

Study design, sample, and procedures

This study was designed as an observational, cross-sectional study conducted among physicians in Montenegro. The study population included both specialist and non-specialist physicians employed at tertiary, secondary, and primary healthcare institutions.

The required sample size was initially estimated using G*Power 3.1.2 (developed by Faul et al.; Heinrich Heine University Düsseldorf, Düsseldorf, Germany) [23] based on the prevalence objective of the study. Additionally, an a priori power analysis was conducted for the multiple regression models using a fixed model (R² deviation from zero), assuming a medium effect size (f² = 0.15), a significance level of α = 0.05, statistical power of 0.95, and 11 predictors. The analysis indicated a minimum required sample size of 178 participants. Therefore, the final sample of 235 physicians was considered sufficient for the planned regression analyses.

This study was part of a larger self-funded research project entitled “Factors associated with mental health, work performance, and well-being of healthcare professionals”. The aim of the project was to investigate the mental health of healthcare professionals, with a particular focus on alexithymia, mentalizing capacity, and resilience as key psychological factors influencing professional burnout, work performance, and physician well-being. The project was approved by the Institutional Review Board for Ethical Evaluation of the University of Belgrade, Faculty of Philosophy, Department of Psychology, Serbia (approval number: #2024-70, date of approval: 18.11.2024). Ethical approval for data collection in Montenegro was obtained from all relevant institutions prior to the study, and the study was conducted in accordance with the principles of the Declaration of Helsinki [24] and Good Clinical Practice guidelines. Participants provided informed consent after receiving detailed explanations regarding the study purpose, procedures, potential risks and benefits, voluntary participation, and confidentiality of responses.

Eligible participants were licensed physicians actively engaged in clinical practice at the primary, secondary, or tertiary healthcare level who provided written informed consent. Healthcare professionals not directly involved in patient care and non-physician staff were excluded from the study. All participants were informed of their right to withdraw from the study at any time without any consequences. 

The study was conducted using a paper-and-pencil survey method and included 235 participants. A total of 270 questionnaires were distributed, of which 235 were completed fully and correctly and were included in the final analysis. Ten participants completed the questionnaire incorrectly, while the remaining individuals declined to participate. The study was conducted between November 3 and November 28, 2025.

All participants provided written informed consent prior to participation. Confidentiality and anonymity were ensured throughout the study.

Instruments

Since validated Montenegrin versions of the study instruments are not available, validated Serbian versions were used. Serbian is widely understood and routinely used in healthcare and academic settings in Montenegro, making these questionnaires linguistically appropriate for physicians in the present study.

Burnout was assessed using the Serbian version of the Maslach Burnout Inventory-Human Services Survey (MBI-HSS) [4]. The instrument consists of 22 items scored on a 7-point Likert scale ranging from 0 (never) to 6 (every day) and assesses three dimensions of burnout: emotional exhaustion (nine items, score range 0-54), depersonalization (five items, score range 0-30), and personal accomplishment (eight items, score range 0-48). Higher scores on the emotional exhaustion and depersonalization subscales indicate higher levels of burnout, whereas lower scores on the personal accomplishment subscale indicate greater burnout. Continuous scores for each dimension were used in the analyses.

Alexithymia was measured using the 20-item Toronto Alexithymia Scale (TAS-20), a widely used instrument in both clinical and non-clinical research [9,10]. The TAS-20 comprises three subscales: difficulty identifying feelings, difficulty describing feelings, and externally oriented thinking. A total score ranging from 20 to 100 is obtained by summing all items. Higher scores indicate greater difficulty in identifying, describing, and processing emotions.

Mentalization capacity was assessed using the RFQ-8, a short version of the original 46-item instrument [14]. The RFQ-8 consists of eight items rated on a 7-point Likert scale ranging from 1 (strongly disagree) to 7 (strongly agree) and assesses the self-reported ability to understand one’s own and others’ mental states. The instrument comprises two four-item subscales: Certainty about Mental States (RFQ-C; 4 items) and Uncertainty about Mental States (RFQ-U; 4 items). Following the standard RFQ scoring procedure described by Fonagy et al. [14], item responses were recoded to generate transformed mean subscale scores ranging from 0 to 3. Higher RFQ-U scores indicate greater uncertainty about mental states (hypomentalizing), whereas higher RFQ-C scores indicate greater certainty about mental states [25].

Sociodemographic and work-related variables were assessed using a specifically designed section of the questionnaire. Sociodemographic data included gender, age, marital status, and number of children. Work-related variables included profession (non-specialist physician, specialist physician), years of work experience, and type of healthcare institution (primary, secondary, tertiary level). The English translation of the questionnaire is presented in Appendix A.

Data analyses

Statistical analyses were performed using IBM SPSS Statistics, version 21.0 (IBM Corp., Armonk, NY, USA). Descriptive statistics were used to summarize the data, including frequencies and percentages for categorical variables and means, standard deviations, minimum and maximum values, skewness, and kurtosis for continuous variables.

The distribution of variables was evaluated using the Kolmogorov-Smirnov and Shapiro-Wilk tests. As the data did not meet the assumptions of normality, nonparametric statistical methods were employed. Group differences were examined using the Mann-Whitney U test and the Kruskal-Wallis test, followed by Bonferroni-adjusted post hoc comparisons when applicable. Relationships between continuous variables were analyzed using Spearman’s rank-order correlation coefficient.

To examine the independent contributions of sociodemographic, work-related, and psychological variables to burnout dimensions (emotional exhaustion, depersonalization, and personal accomplishment), hierarchical linear regression analyses were conducted. In the first step, sociodemographic and work-related variables were entered as control variables. In the second step, psychological variables (alexithymia, hypomentalizing, and hypermentalizing) were added. Although means and standard deviations are presented for descriptive purposes, given the observed deviations from normality, subsequent analyses and group comparisons were conducted using nonparametric methods and are therefore reported using medians and interquartile ranges where appropriate. Despite these deviations, hierarchical linear regression was applied to assess the independent contributions of predictor variables. Regression assumptions were evaluated using residual diagnostics, including inspection of standardized residuals, normal probability plots, residual scatterplots, and multicollinearity statistics. Potential outliers were examined using standard diagnostic procedures, and no evidence of influential cases affecting model stability was identified. To further evaluate the robustness of the regression estimates in the presence of non-normality, bootstrap analyses with 5,000 resamples and bias-corrected accelerated (BCa) 95% confidence intervals were additionally performed for all regression models.

Reliability of the instruments was assessed using Cronbach’s alpha coefficient. All statistical tests were two-tailed, and a p-value < 0.05 was considered statistically significant.

## Results

Participant characteristics

The sociodemographic and work-related characteristics of the respondents are presented in Table 1. A total of 235 physicians participated in the study. The majority of respondents were female (62.1%), while males accounted for 37.9% of the sample. The average age of participants was 43.44 ± 11.30 years.

**Table 1:** Sociodemographic and work-related characteristics of the study participants (N = 235) Continuous variables are presented as mean ± standard deviation (SD), and categorical variables are presented as n (%).

Characteristics	Value
Age (years)	43.44 ± 11.30
Gender	
Male	89 (37.9)
Female	146 (62.1)
Profession	
Non-specialist physician	87 (37.0)
Specialist physician	148 (63.0)
Years of work experience	15.74 ± 10.42
Type of healthcare institution	
Primary level	90 (38.3)
Secondary level	48 (20.4)
Tertiary level	97 (41.3)
Marital status	
Married/cohabiting	126 (53.6)
Not married/not cohabiting	109 (46.4)
Number of children	
No children	88 (37.4)
One child	38 (16.2)
Two or more children	109 (46.4)

Regarding profession, most participants were specialist physicians (63.0%), whereas 37.0% were non-specialist physicians. The average work experience was 15.74 ± 10.42 years. In terms of the type of healthcare institution, most respondents were employed at the tertiary level (41.3%), followed by the primary (38.3%) and secondary levels (20.4%). Regarding marital status, more than half of the respondents were married or cohabiting (53.6%), while 46.4% were not married/not cohabiting. With respect to family characteristics, 37.4% of participants had no children, 16.2% had one child, while the largest proportion (46.4%) reported having two or more children.

Descriptive statistics of mentalizing, alexithymia, and burnout dimensions

Table 2 shows the descriptive statistics and reliability of the applied scales. Inspection of skewness and kurtosis indicated deviations from normality, particularly for depersonalization and uncertainty about mental states (RFQ-U). Therefore, nonparametric methods were used for group comparisons and correlation analyses. Internal consistency ranged from borderline to excellent, with Cronbach’s alpha coefficients ranging from 0.68 to 0.94 and the personal accomplishment subscale showing the lowest reliability (α = 0.68).

**Table 2:** Descriptive statistics and reliability of the applied scales Min.: minimum; Max.: maximum; SD: standard deviation; IQR: interquartile range; Skew: skewness; Kurt: kurtosis; α: Cronbach's alpha; EE: emotional exhaustion; DP: depersonalization; PA: personal accomplishment; RFQ-U: Reflective Functioning Questionnaire (uncertainty, hypomentalizing); RFQ-C: Reflective Functioning Questionnaire (certainty, hypermentalizing); TAS-20: Toronto Alexithymia Scale

Scale	Min.	Max.	Mean	SD	Median	IQR	Skew	Kurt	α
EE	0.00	54.00	37.28	12.47	41.00	17.00	-0.97	0.14	0.94
DP	0.00	48.00	9.00	6.56	8.00	9.00	1.30	4.41	0.73
PA	10.00	47.00	35.37	5.31	36.00	7.00	-0.72	2.14	0.68
RFQ-U	0.00	3.00	0.48	0.62	0.25	0.75	1.50	1.82	0.70
RFQ-C	0.00	2.75	0.72	0.73	0.50	1.25	0.87	-0.26	0.72
TAS-20	24.00	100.00	57.32	13.08	55.00	15.00	0.73	0.56	0.86

Relationship among mentalizing, alexithymia, and burnout dimensions

Table 3 presents the differences in burnout dimensions according to sociodemographic and work-related characteristics. Statistically significant differences in all three burnout dimensions were observed between male and female participants. Male participants showed higher levels of emotional exhaustion and depersonalization (p < 0.05), whereas female participants had higher levels of personal accomplishment (p < 0.01). Age and years of work experience were positively correlated with emotional exhaustion (p < 0.001). In addition, age was weakly but significantly associated with depersonalization (p < 0.05), while years of work experience were not significantly associated with depersonalization. Neither age nor work experience was significantly associated with personal accomplishment (p > 0.05). Regarding professional status, physicians without specialization had lower levels of emotional exhaustion compared to specialist physicians (p < 0.001), whereas no significant differences were observed for depersonalization and personal accomplishment (p > 0.05). Significant differences in emotional exhaustion (p < 0.001) and depersonalization (p < 0.05) were observed across levels of healthcare institutions. Post hoc analysis indicated that participants working at the tertiary level had higher values of these burnout dimensions compared to those at the primary level. No significant differences were found in personal accomplishment across institutions. Marital status was not significantly associated with any burnout dimension. Although a statistically significant difference in emotional exhaustion was initially observed across categories of number of children (p < 0.05), this association was not retained after Bonferroni correction. No differences were found for depersonalization and personal accomplishment.

**Table 3:** Associations of sociodemographic and work-related characteristics with burnout dimensions Data are presented as median (interquartile range, IQR) or Spearman's correlation coefficient (ρ). Between-group differences were analyzed using the Mann–Whitney U test (*) and Kruskal–Wallis test (#), while associations with continuous variables were assessed using Spearman's rank correlation (†), as appropriate. Effect size is presented as the rank-biserial correlation (r<sub>rb</sub>) for Mann–Whitney U tests and epsilon-squared (ε²) for Kruskal–Wallis tests. Superscript letters (a, b) indicate the results of post hoc pairwise comparisons: groups sharing the same superscript letter do not differ significantly, whereas groups with different superscript letters differ significantly. Although the omnibus Kruskal–Wallis test indicated a significant difference in emotional exhaustion according to the number of children (p = 0.042), none of the post hoc pairwise comparisons remained statistically significant after Bonferroni correction. EE: emotional exhaustion; DP: depersonalization; PA: personal accomplishment; χ²: chi-square

Variable	Category	EE Median (IQR)/ρ	Test statistic	p	Effect size	DP Median (IQR)/ρ	Test statistic	p	Effect size	PA Median (IQR)/ρ	Test statistic	p	Effect size
Gender	Male	43.00 (14.00)	U = 5231.0	0.012*	0.2	11.00 (9.00)	U = 4475.5	<0.001*	0.31	35.00 (6.00)	U = 5109.0	0.006*	0.21
	Female	38.50 (19.25)				6.00 (9.00)				36.00 (6.00)			
Age (years)		0.397		<0.001†		0.161		0.013†		0.032		0.630†	
Profession	Physicians without specialization	33.00 (19.00)	U = 3950.5	<0.001*	0.39	6.00 (10.00)	U = 5822.5	0.220*	0.1	36.00 (8.00)	U = 6244.0	0.699*	0.03
	Specialist physicians	44.00 (12.00)				8.50 (9.75)				36.00 (5.00)			
Years of work experience		0.293		<0.001†		0.101		0.122†		0.025		0.706†	
Type of healthcare institution	Primary	35.50 (20.00)^a^	χ² = 18.1	<0.001^#^	0.07	6.00 (9.00)ᵃ	χ² = 6.2	0.045^#^	0.02	36.00 (7.00)	χ² = 0.6	0.746^#^	0.00
	Secondary	42.00 (13.75)^ab^				8.00 (7.00)ᵃᵇ				35.50 (6.00)			
	Tertiary	44.00 (12.00)^b^				10.00 (11.00)ᵇ				36.00 (5.50)			
Marital status	Married/cohabiting	42.00 (18.00)	U = 6650.5	0.677*	0.03	7.00 (9.00)	U = 6277.0	0.255*	0.09	36.00 (7.00)	U = 6543.0	0.532*	0.05
	Single	41.00 (16.00)				8.00 (10.50)				36.00 (6.00)			
Number of children	No children	37.00 (16.00)	χ² = 6.3	0.042^#^	0.02	8.00 (10.00)	χ² = 0.1	0.943^#^	0.00	36.00 (7.00)	χ² = 1.1	0.586^#^	0.00
	One child	41.50 (20.50)				8.50 (11.00)				36.00 (8.25)			
	Two or more children	43.00 (15.00)				7.00 (9.00)				36.00 (6.00)			

The correlations between burnout dimensions, mentalizing capacity, and alexithymia are presented in Table 4. Emotional exhaustion was positively correlated with depersonalization (ρ = 0.56; p < 0.001) and hypomentalizing (ρ = 0.27; p < 0.001) and negatively correlated with hypermentalizing (ρ = -0.51; p < 0.001). No significant associations were observed between emotional exhaustion and personal accomplishment or alexithymia (p > 0.05).

**Table 4:** Spearman correlations between burnout dimensions, mentalization and alexithymia Spearman’s correlation coefficients are presented. * p < 0.05; ** p < 0.01; *** p < 0.001. EE: emotional exhaustion; DP: depersonalization; PA: personal accomplishment; RFQ-U: Reflective Functioning Questionnaire (uncertainty, hypomentalizing); RFQ-C: Reflective Functioning Questionnaire (certainty, hypermentalizing); TAS-20: Toronto Alexithymia Scale.

Variable	EE	DP	PA	RFQ-u	RFQ-c	TAS-20
EE	1					
DP	0.56***	1				
PA	-0.08	-0.35***	1			
RFQ-U	0.27***	0.17**	-0.01	1		
RFQ-C	-0.51***	-0.33***	0.12	-0.43***	1	
TAS-20	0.13	0.10	-0.13	0.53***	-0.41***	1

Depersonalization was negatively correlated with personal accomplishment (ρ = -0.35; p < 0.001) and hypermentalizing (ρ = -0.33; p < 0.001) and positively correlated with hypomentalizing (ρ = 0.17; p < 0.05). No significant association was found between depersonalization and alexithymia (p > 0.05).

Hypomentalizing was positively correlated with alexithymia (ρ = 0.53; p < 0.001). In contrast, hypermentalizing was negatively correlated with both hypomentalizing (ρ = -0.43; p < 0.001) and alexithymia (ρ = -0.41; p < 0.001).

No statistically significant associations were observed between personal accomplishment and the examined mentalization dimensions or alexithymia (p > 0.05).

Prior to performing the hierarchical regression analyses, key statistical assumptions were evaluated. The results confirmed that the assumptions of normality, linearity, homoscedasticity, and absence of multicollinearity were satisfactorily fulfilled. Multicollinearity was not considered problematic, as variance inflation factor (VIF) values for all variables remained below the recommended cutoff of 5. The Durbin-Watson statistics were 1.470 for emotional exhaustion, 1.582 for depersonalization, and 2.038 for personal accomplishment, suggesting no meaningful autocorrelation of residuals. Due to elevated multicollinearity between age and years of work experience observed in preliminary analyses (VIF > 5), only age was retained in the final regression models to minimize redundancy and enhance model stability and interpretability [26].

The results presented in Table 5 indicate that sociodemographic and work-related variables accounted for a significant amount of explained variance in emotional exhaustion at the first step of the model (R2 = 0.182). Within this set of variables, age showed a positive association with emotional exhaustion (β = 0.38; p < 0.001), whereas no statistically significant relationships were identified for gender, profession, marital status, number of children, or type of healthcare institution.

**Table 5:** Hierarchical linear regression analysis predicting emotional exhaustion Standardized beta coefficients (β), t-values, variance inflation factors (VIF), and 95% confidence intervals for the unstandardized regression coefficients (95% CI for B) are presented. A dash (–) indicates that the variable was not included in the corresponding regression model. R²: coefficient of determination; Adjusted R²: adjusted coefficient of determination; ΔR²: change in the coefficient of determination between Model 1 and Model 2; F change: change in F statistic; * p < 0.05; ** p < 0.01; *** p < 0.001. Gender was coded as 0 = male and 1 = female. Profession was coded as 0 = physicians without specialization and 1 = specialist physicians. Reference categories were the primary level of healthcare for level of healthcare, married for marital status, and no children for number of children. RFQ-U: Reflective Functioning Questionnaire (uncertainty, hypomentalizing); RFQ-C: Reflective Functioning Questionnaire (certainty, hypermentalizing); TAS-20: Toronto Alexithymia Scale.

Variable	Model 1 (Control variables)				Model 2 (Control variables + predictors)			
	β	t	VIF	95% CI for B	β	t	VIF	95% CI for B
Gender	-0.12	-1.86	1.06	-6.075 to 0.178	-0.05	-0.87	1.09	-3.943 to 1.521
Age	0.38***	3.97	2.48	0.209 to 0.621	0.29***	3.54	2.56	0.144 to 0.505
Profession	-0.06	-0.60	2.71	-6.550 to 3.513	-0.09	-1.06	2.78	-6.762 to 2.024
Secondary level	0.12	1.57	1.63	-0.960 to 8.371	0.15*	2.21	1.63	0.484 to 8.546
Tertiary level	0.15	1.74	1.95	-0.487 to 7.882	0.12	1.64	1.97	-0.602 to 6.647
Marital status	-0.01	-0.14	1.53	-3.909 to 3.406	-0.01	-0.13	1.54	-3.376 to 2.946
One child	-0.02	-0.26	1.55	-5.641 to 4.330	0.02	0.35	1.56	-3.558 to 5.071
Two or more children	-0.05	-0.52	2.30	-5.673 to 3.295	-0.05	-0.61	2.31	-5.080 to 2.673
RFQ-U	–	–	–	–	0.17**	2.66	1.51	0.877 to 5.910
RFQ-C	–	–	–	–	-0.47***	-7.96	1.31	-10.095 to -6.089
TAS-20	–	–	–	–	-0.20**	-3.02	1.58	-0.310 to -0.065
R^2^	0.182				0.400			
Adjusted R^2^	0.153				0.370			
F change	6.29***				26.97***			
ΔR^2^	0.218***							

After the inclusion of psychological variables in the second step, the model showed a substantial increase in explained variance (R2 = 0.400; ΔR2 = 0.218; p < 0.001). Age remained a significant positive predictor of emotional exhaustion (β = 0.29; p < 0.001). In addition, working at the secondary level of healthcare was associated with higher emotional exhaustion compared with the primary level (β = 0.15; p < 0.05).

Among psychological variables, higher levels of hypomentalizing were associated with higher emotional exhaustion (β = 0.17; p < 0.05), whereas higher levels of hypermentalizing were associated with lower emotional exhaustion (β = -0.47; p < 0.001). Alexithymia was also negatively associated with emotional exhaustion (β = -0.20; p < 0.01).

The results presented in Table 6 showed that sociodemographic and work-related variables explained a significant proportion of the variance in depersonalization in the initial model (R² = 0.115). Within this model, both gender and age emerged as significant predictors, with higher levels of depersonalization observed among male participants (β = -0.24; p < 0.001) and older individuals (β = 0.25; p < 0.05). No significant effects were found for profession, marital status, number of children, or type of healthcare institution.

**Table 6:** Hierarchical linear regression analysis predicting depersonalization Standardized beta coefficients (β), t-values, variance inflation factors (VIF), and 95% confidence intervals for the unstandardized regression coefficients (95% CI for B) are presented. A dash (–) indicates that the variable was not included in the corresponding regression model. R²: coefficient of determination; Adjusted R²: adjusted coefficient of determination; ΔR²: change in the coefficient of determination between Model 1 and Model 2; F change: change in F statistic; * p < 0.05; ** p < 0.01; *** p < 0.001. Gender was coded as 0 = male and 1 = female. Profession was coded as 0 = physicians without specialization and 1 = specialist physicians. Reference categories were the primary level of healthcare for level of healthcare, married for marital status, and no children for number of children. RFQ-U: Reflective Functioning Questionnaire (uncertainty, hypomentalizing); RFQ-C: Reflective Functioning Questionnaire (certainty, hypermentalizing); TAS-20: Toronto Alexithymia Scale.

Variable	Model 1 (Control variables)				Model 2 (Control variables + predictors)			
	β	t	VIF	95% CI for B	β	t	VIF	95% CI for B
Gender	-0.24***	-3.73	1.06	-4.949 to -1.529	-0.19**	-3.12	1.09	-4.273 to -0.966
Age	0.25*	2.51	2.48	0.031 to 0.256	0.20*	2.14	2.56	0.009 to 0.228
Profession	-0.16	-1.56	2.71	-4.929 to 0.575	-0.19	-1.86	2.78	-5.165 to 0.151
Secondary level	0.01	0.11	1.63	-2.412 to 2.692	0.02	0.30	1.63	-2.063 to 2.815
Tertiary level	0.10	1.11	1.95	-0.995 to 3.583	0.08	0.99	1.97	-1.089 to 3.297
Marital status	0.06	0.76	1.53	-1.234 to 2.766	0.05	0.74	1.54	-1.197 to 2.628
One child	-0.06	-0.71	1.55	-3.704 to 1.750	-0.03	-0.43	1.56	-3.178 to 2.044
Two or more children	-0.10	-1.07	2.30	-3.783 to 1.122	-0.10	-1.14	2.31	-3.698 to 0.993
RFQ-U	–	–	–	–	0.19*	2.54	1.51	0.436 to 3.481
RFQ-C	–	–	–	–	-0.26***	-3.80	1.31	-3.551 to -1.127
TAS-20	–	–	–	–	-0.15	-1.95	1.58	-0.147 to 0.001
R^2^	0.115				0.206			
Adjusted R^2^	0.084				0.166			
F change	3.68***				8.44***			
ΔR^2^	0.091***							

After the inclusion of psychological variables in the second step, the model showed an increase in explained variance (R2 = 0.206; ΔR2 = 0.091; p < 0.001). Gender and age remained significant predictors of depersonalization.

Among psychological variables, higher levels of hypomentalizing were associated with higher depersonalization (β = 0.19, p < 0.05), whereas higher levels of hypermentalizing were associated with lower depersonalization (β = −0.26, p < 0.001). Alexithymia was not significantly associated with depersonalization in the final model, although the association approached the conventional threshold for statistical significance (β = −0.15, p = 0.053).

The results presented in Table 7 showed that hierarchical regression analysis did not identify sociodemographic and work-related variables entered in the first step, nor psychological variables added in the second step, as significant predictors of personal accomplishment. Although the inclusion of psychological variables resulted in a small increase in explained variance (ΔR² = 0.022), the overall model remained non-significant. Therefore, no further interpretation of individual predictors was undertaken.

**Table 7:** Hierarchical linear regression analysis predicting personal accomplishment Standardized beta coefficients (β), t-values, variance inflation factors (VIF), and 95% confidence intervals for the unstandardized regression coefficients (95% CI for B) are presented. A dash (–) indicates that the variable was not included in the corresponding regression model. R²: coefficient of determination; Adjusted R²: adjusted coefficient of determination; ΔR²: change in the coefficient of determination between Model 1 and Model 2; F change: change in F statistic; * p < 0.05; ** p < 0.01; *** p < 0.001. Gender was coded as 0 = male and 1 = female. Profession was coded as 0 = physicians without specialization and 1 = specialist physicians. Reference categories were the primary level of healthcare for level of healthcare, married for marital status, and no children for number of children. RFQ-U: Reflective Functioning Questionnaire (uncertainty, hypomentalizing); RFQ-C: Reflective Functioning Questionnaire (certainty, hypermentalizing); TAS-20: Twenty-Item Toronto Alexithymia Scale.

Variable	Model 1 (Control variables)				Model 2 (Control variables + predictors)			
	β	t	VIF	95% CI for B	β	t	VIF	95% CI for B
Gender	0.17	2.58	1.06	0.446 to 3.318	0.16	2.33	1.09	0.263 to 3.159
Age	0.05	0.52	2.48	−0.070 to 0.120	0.09	0.90	2.56	−0.052 to 0.139
Profession	-0.04	-0.39	2.71	−2.768 to 1.854	-0.07	-0.65	2.78	−3.102 to 1.555
Secondary level	-0.04	-0.52	1.63	−2.712 to 1.575	-0.04	-0.51	1.63	−2.693 to 1.580
Tertiary level	0.05	0.51	1.95	−1.422 to 2.423	0.07	0.72	1.97	−1.216 to 2.627
Marital status	-0.03	-0.32	1.53	−1.957 to 1.403	-0.02	-0.30	1.54	−1.929 to 1.422
One child	0.10	1.20	1.55	−0.898 to 3.683	0.09	1.15	1.56	−0.957 to 3.617
Two or more children	0.08	0.84	2.30	−1.183 to 2.937	0.07	0.70	2.31	−1.324 to 2.786
RFQ-U	–	–	–	–	0.05	0.61	–	−0.918 to 1.750
RFQ-C	–	–	–	–	0.08	1.04	–	−0.503 to 1.620
TAS-20	–	–	–	–	-0.13	-1.62	–	−0.118 to 0.011
R^2^	0.048				0.070			
Adjusted R^2^	0.014				0.024			
F change	1.42				1.74			
ΔR^2^	0.022							

## Discussion

Building upon the theoretical framework presented earlier, this study focused on exploring how sociodemographic factors, alexithymia, and mentalizing capacity are associated with various dimensions of burnout among physicians in Montenegro, as well as on assessing their independent contributions after controlling for relevant factors. The findings underscore the significant contribution of psychological factors, particularly mentalizing capacity, in understanding burnout among physicians. These findings should be interpreted in a broader international context, as burnout among physicians is reported to be high in Western European and American countries, while additional structural and organizational challenges characteristic of healthcare systems in transition countries may further contribute to this burden [27]. In line with findings from regional studies conducted in healthcare settings in the Balkans, where mentalizing capacity was also identified as an important factor associated with burnout among healthcare workers, the results of this study further support the relevance of psychological resources in shaping the outcome of work-related stress [28].

The healthcare system in Montenegro is characterized by limited resources, chronic labor shortages, high administrative burden, and frequent shift work, all of which may contribute to increased work-related stress and burnout risk [22]. Consistent with contemporary models that conceptualize burnout as the result of an interaction between individual psychological characteristics and work-related conditions [29] and which highlight the role of mentalizing processes in emotional regulation and interpersonal functioning [30], the present findings indicate that different dimensions of burnout are differentially associated with the examined factors.

The findings of the present study are consistent with previous research, as reported by Safiye et al., indicating that hypomentalizing is associated with higher levels of emotional exhaustion and depersonalization, as well as lower levels of personal accomplishment [28]. Similarly, in our study, hypomentalizing emerged as a significant positive predictor of emotional exhaustion and depersonalization, whereas higher RFQ-C scores were associated with lower levels of emotional exhaustion and depersonalization. These findings may be interpreted in light of contemporary mentalizing theory, which emphasizes the importance of understanding one's own and others' mental states for emotional regulation and adaptive interpersonal functioning, particularly in demanding clinical settings [30]. Reduced mentalizing capacity may be associated with difficulties in interpreting emotional experiences, which may in turn be associated with greater interpersonal strain, professional frustration, and emotional burden [30]. In contrast, greater certainty in understanding mental states may be associated with more effective emotional regulation and adaptive coping with occupational stress [31].

However, the interpretation of RFQ-C requires particular caution. Although higher RFQ-C scores are frequently interpreted as reflecting hypermentalizing, hypermentalizing itself is generally conceptualized as a biased or maladaptive form of mentalizing rather than a protective characteristic [20]. In non-clinical occupational samples such as the present one, higher RFQ-C scores may instead reflect greater certainty in understanding mental states or more adaptive reflective functioning, rather than pathological overmentalizing. Therefore, the observed inverse association between RFQ-C scores and burnout should not be interpreted as evidence that hypermentalizing is protective. Rather, it may reflect greater confidence in understanding one's own and others' mental states, which in this non-clinical occupational sample was associated with lower burnout among physicians. Given the ongoing conceptual debate regarding the interpretation of RFQ-C and the recognized limitations of the RFQ-8 in distinguishing adaptive certainty from maladaptive overconfidence, these findings should be interpreted with caution and require further investigation [20]. Accordingly, the present findings should be interpreted in terms of RFQ-C scores rather than as evidence for beneficial effects of hypermentalizing itself. Importantly, the use of the RFQ in a non-clinical sample of healthcare professionals represents an innovative approach, suggesting that mentalizing may be understood not only as a psychopathological construct but also as a psychological characteristic relevant to professional functioning in clinical practice [20].

An additional important finding is the association between alexithymia and mentalizing. Specifically, hypomentalizing showed a strong positive association with alexithymia, whereas higher RFQ-C scores were negatively associated with this construct. These findings are consistent with previous research suggesting that difficulties in identifying and describing emotions directly impair mentalizing capacity [32, 33, 34].

In the univariate analysis, alexithymia was not significantly associated with any dimension of burnout. However, in the multivariate model, it emerged as a significant predictor of emotional exhaustion, while no significant associations were observed with depersonalization or personal accomplishment. In contrast to previous evidence, which has consistently demonstrated that alexithymia is associated with increased emotional exhaustion and depersonalization, together with reduced personal accomplishment, the present findings only partially correspond to these established patterns [11, 35]. Furthermore, alexithymia has been linked to a broader spectrum of psychological difficulties, including increased stress, anxiety, and depressive symptoms, which may further elevate the risk of burnout among healthcare professionals [36]. Additional sequential regression analyses showed that the regression coefficient for alexithymia changed progressively after the inclusion of hypomentalizing and hypermentalizing variables, shifting from a small positive, non-significant association to a significant negative association with emotional exhaustion. This pattern is consistent with a possible suppression effect, suggesting that the shared variance between alexithymia and mentalizing capacity dimensions may have obscured the unique association between alexithymia and emotional exhaustion in the univariate analysis [37]. However, because suppression was not formally tested using dedicated statistical procedures, this interpretation should be considered exploratory. Overall, these findings suggest that the relationship between alexithymia, mentalizing capacity, and burnout is likely to be more complex than a simple direct association, and future studies employing formal suppression, mediation, or path analyses are needed to clarify these underlying mechanisms.

When considering sociodemographic and occupational variables, the results highlight age and gender as relevant determinants of specific burnout dimensions. In particular, higher levels of emotional exhaustion and depersonalization were observed among older participants. This finding contrasts with studies reporting higher burnout among younger individuals, often attributed to less developed coping mechanisms and lower emotional resilience [38]. A possible explanation for this pattern is that, within the medical profession, prolonged exposure to occupational stress, high demands, and responsibility may be associated with a cumulative burden of professional strain over time [39]. This finding may also be interpreted within the framework of compassion fatigue, in which long-term exposure to emotionally demanding clinical environments has been associated with gradual depletion of emotional resources and reduced empathic engagement [39].

This finding may also reflect the fact that senior physicians in Montenegro often assume greater clinical, supervisory, and administrative responsibilities than their younger colleagues, which could further increase their cumulative occupational burden. Differences in healthcare organization, resource availability, cultural expectations, and cohort characteristics may also partly explain why our findings differ from studies reporting higher burnout among younger physicians. Nevertheless, because of the cross-sectional design, these explanations remain hypothetical and should be interpreted with caution.

With regard to gender, male participants reported higher levels of emotional exhaustion and depersonalization, whereas female participants demonstrated higher levels of personal accomplishment. 

One possible explanation is that male physicians in Montenegro may be more frequently employed in certain hospital-based specialties that involve greater clinical responsibility, emergency work, and physically or emotionally demanding tasks, whereas female physicians may be relatively more represented in primary care and other less acutely demanding settings. If such differences in specialty distribution indeed exist, they could partly contribute to the observed gender differences. However, because specialty-specific analyses were not performed in the present study, this explanation should be considered speculative and warrants further investigation.

This finding is not entirely consistent with previous research suggesting that women are more prone to emotional exhaustion, often attributed to greater role strain and challenges in achieving work-life balance [40].

Finally, personal accomplishment was not significantly explained by the examined variables, suggesting that this dimension of burnout may have a distinct psychological basis and may be more strongly related to intrinsic factors such as motivation and professional identity, which reflect individuals’ sense of competence, purpose, and personal investment in their professional role [29].

Limitations and future directions

The present study has several important strengths. To the best of our knowledge, this is the first study to examine the associations between mentalizing capacity, alexithymia, and burnout among physicians in Montenegro. Furthermore, the inclusion of physicians from primary, secondary, and tertiary healthcare settings, together with the use of validated psychometric instruments, provides a comprehensive assessment of psychological factors associated with burnout.

Several limitations need to be taken into account when interpreting the findings. In particular, the cross-sectional design restricts the ability to establish causal relationships. Accordingly, the observed associations should not be interpreted as indicating causal relationships, and reverse or bidirectional associations between burnout and mentalizing capacity cannot be excluded. Second, the use of self-report measures introduces the possibility of response bias. Third, the sample was limited to physicians in Montenegro, which may restrict the generalizability of the findings to other healthcare systems and cultural contexts. Additionally, due to multicollinearity, age and years of work experience could not be included simultaneously in the multivariate models, which may limit the ability to fully disentangle their individual contributions to burnout. Additionally, the study did not include other relevant psychological variables, such as depression, anxiety, or stress, which may be associated with both burnout and emotional processing constructs. Additionally, certain statistical limitations should be acknowledged. Additionally, the internal consistency of the Personal Accomplishment subscale was slightly below the conventional threshold (Cronbach's α = 0.68), which should be considered when interpreting findings related to this burnout dimension. The pronounced skewness observed in depersonalization scores may reflect potential social desirability bias, whereby physicians may be reluctant to report cynical or detached attitudes toward patients. Furthermore, the relatively strong intercorrelations among psychological variables, particularly between alexithymia and mentalization capacity dimensions, may complicate the isolation of their independent effects and should therefore be considered when interpreting the regression findings.

Future studies should employ more advanced analytical approaches, such as structural equation modeling or path analysis, to further elucidate the complex direct and indirect relationships among mentalizing capacity, alexithymia, and burnout dimensions, including potential suppression and mediation mechanisms. Moreover, studies involving larger and more diverse samples across different healthcare systems are warranted.

From a practical perspective, the findings suggest that interventions aimed at enhancing mentalizing capacity and emotional regulation may be worthy of further investigation as potential approaches for addressing burnout among physicians [16]. Such interventions may include Balint groups, which are structured peer discussion groups in which physicians reflect on emotionally challenging clinical encounters and the doctor-patient relationship, as well as peer supervision programs and communication training grounded in mentalization-based treatment principles, which emphasize understanding one's own and others' mental states, strengthening reflective functioning, emotional regulation, and enhancing interpersonal understanding in clinical settings. These findings may have broader implications for staff support programs, undergraduate and postgraduate medical education, and continuing professional development by promoting the integration of mentalizing-informed approaches into training and clinical practice. At the same time, it is important to emphasize that strengthening individual psychological resources should not replace the responsibility of healthcare institutions to address structural and organizational factors contributing to burnout. Efforts to reduce burnout should therefore involve both individual-level interventions and systemic changes, including workload management, organizational support, and improvement of working conditions.

## Conclusions

This study suggests that mentalization capacity is an important psychological factor associated with burnout among physicians in Montenegro. Higher levels of hypomentalizing were associated with greater emotional exhaustion and depersonalization, whereas higher levels of hypermentalizing were associated with lower levels of these burnout dimensions.

The association between alexithymia and burnout should be interpreted cautiously, as the observed findings may reflect a suppression effect rather than a direct relationship.

## Figures and Tables

**Figure 1:**
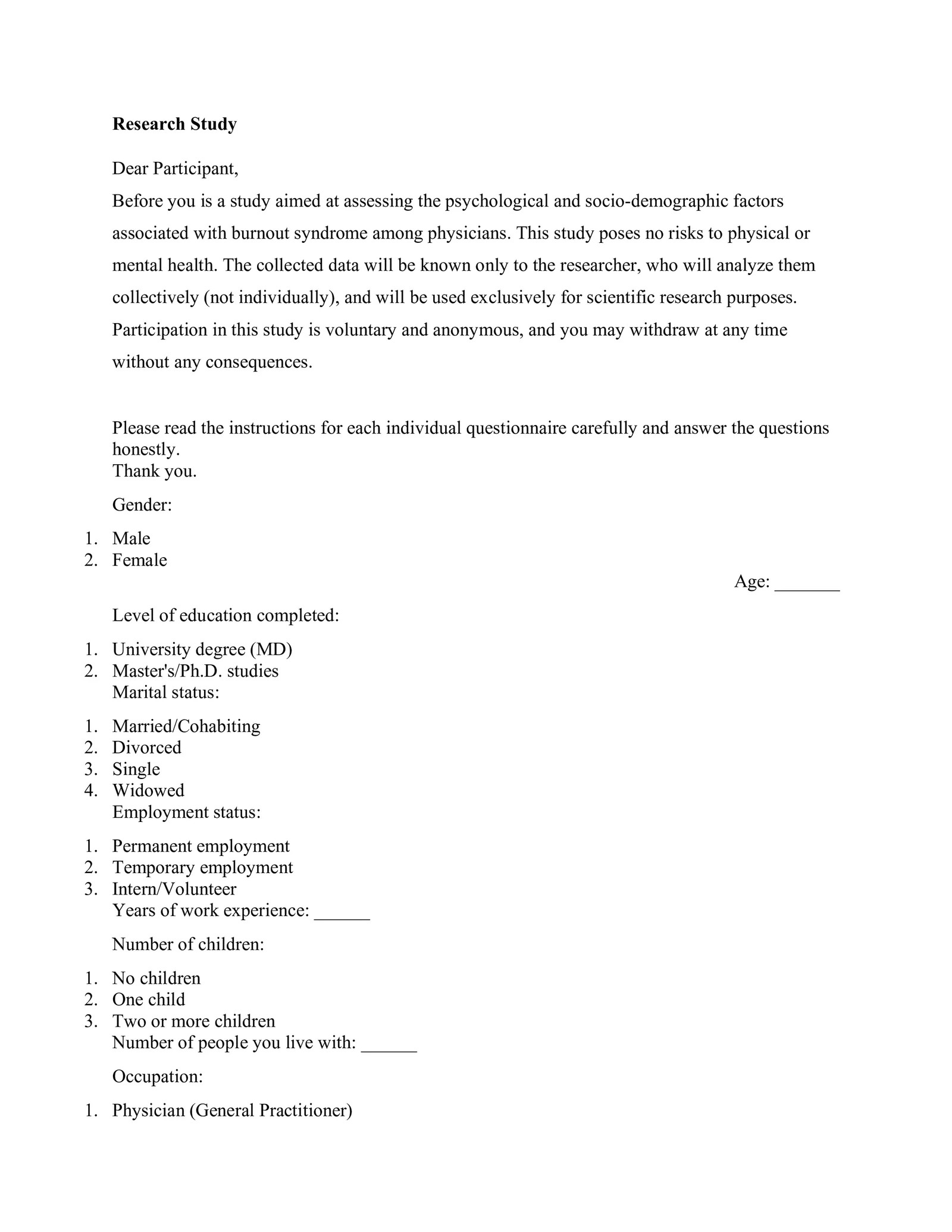
Questionnaire: part 1

**Figure 2:**
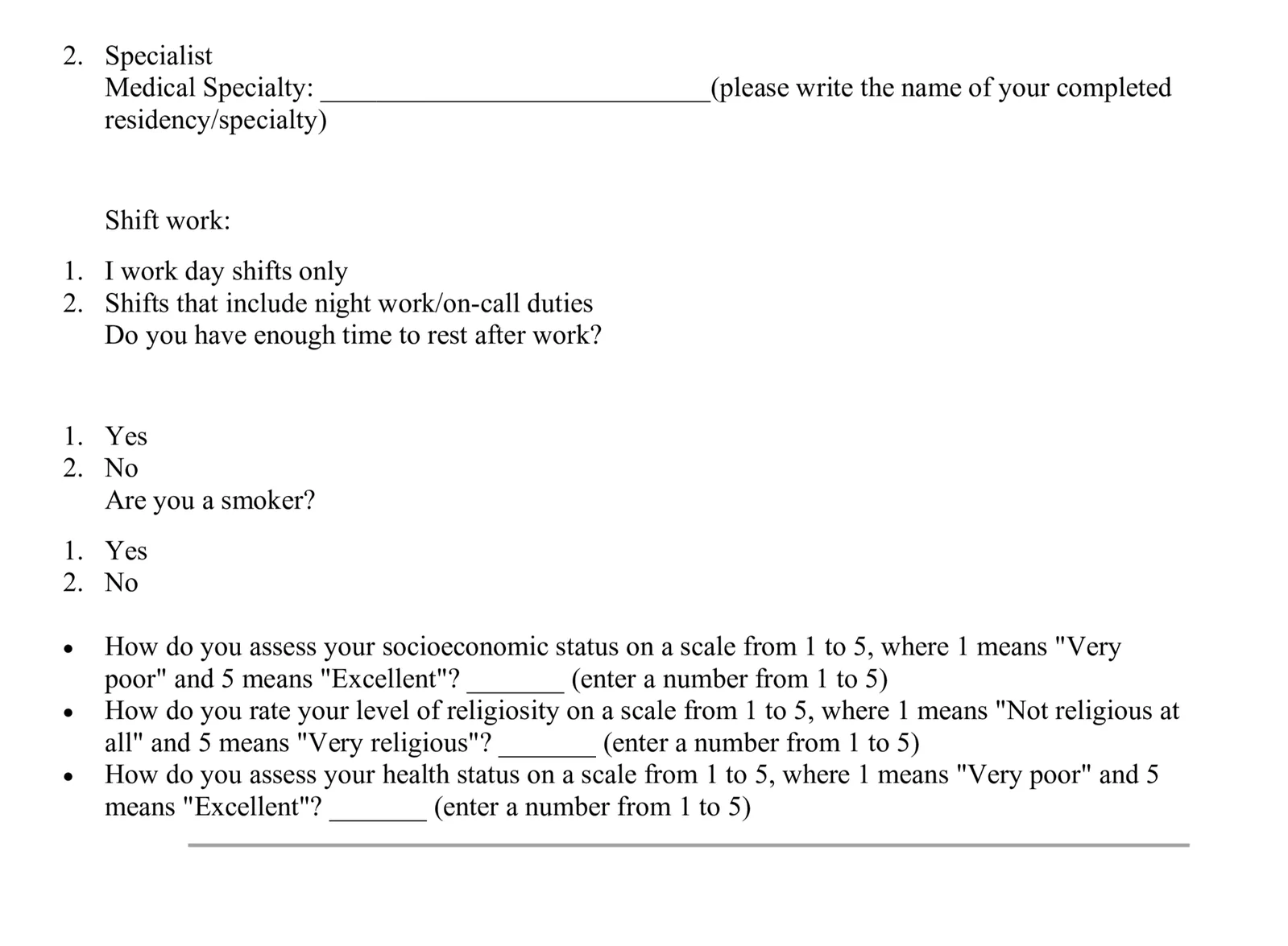
Questionnaire: part 2

**Figure 3:**
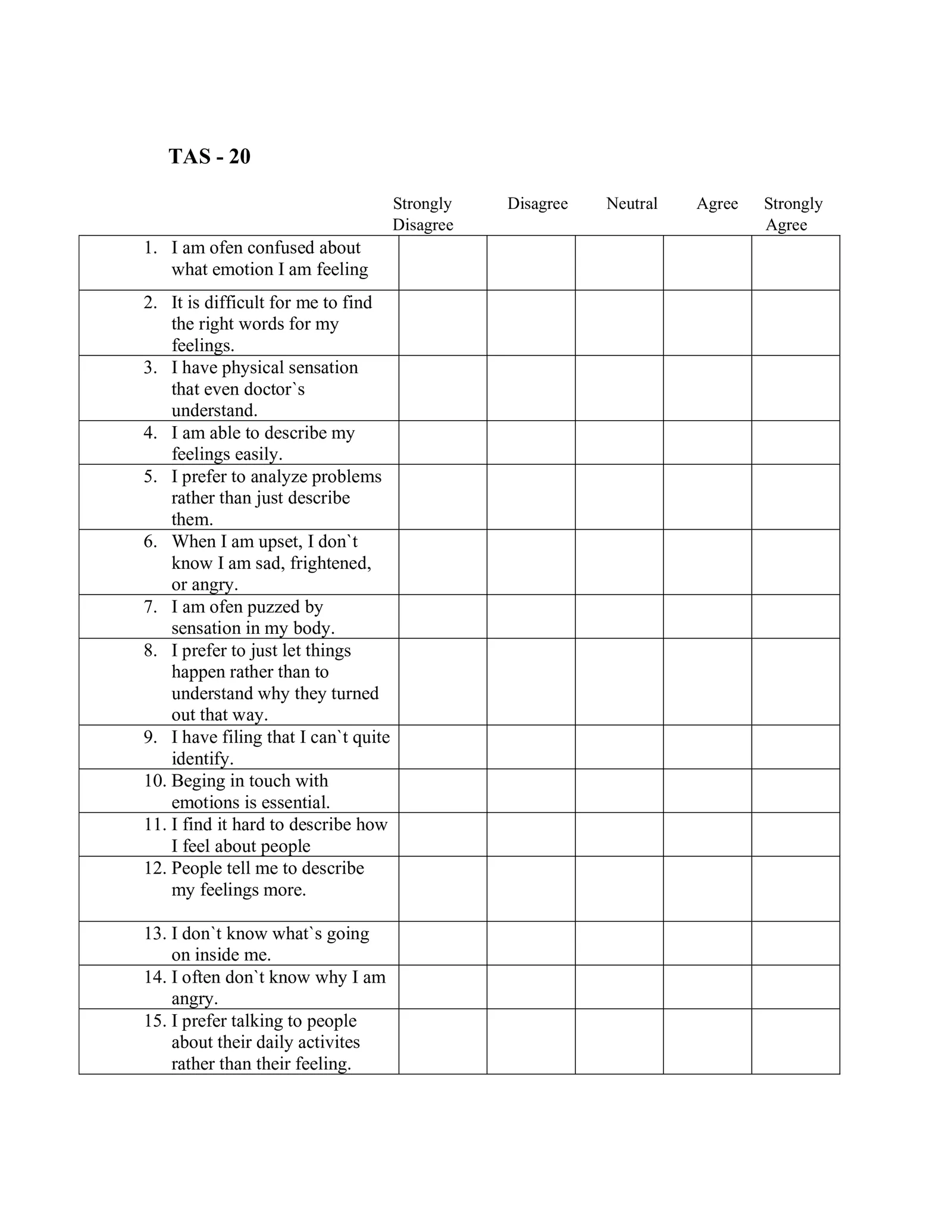
Questionnaire: part 3

**Figure 4:**
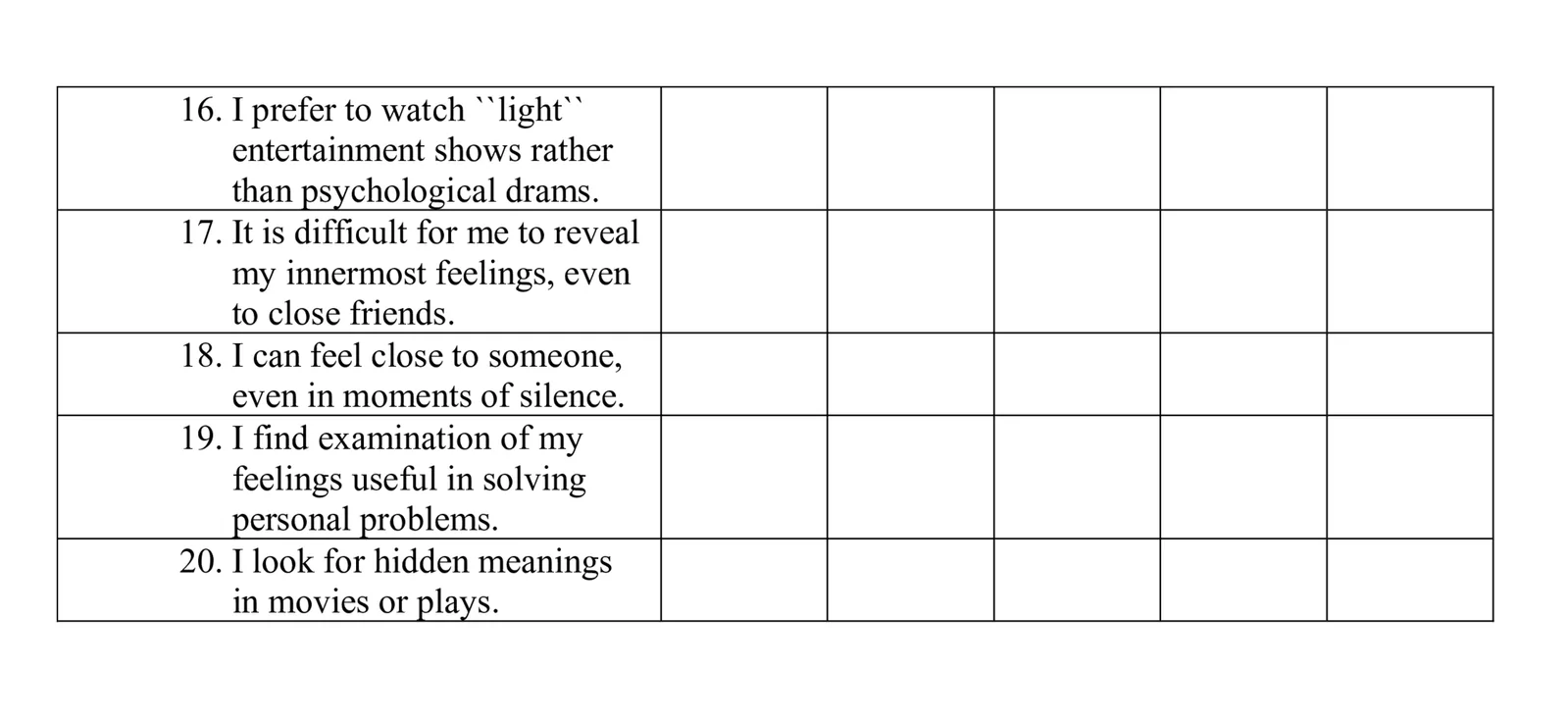
Questionnaire: part 4

**Figure 5:**
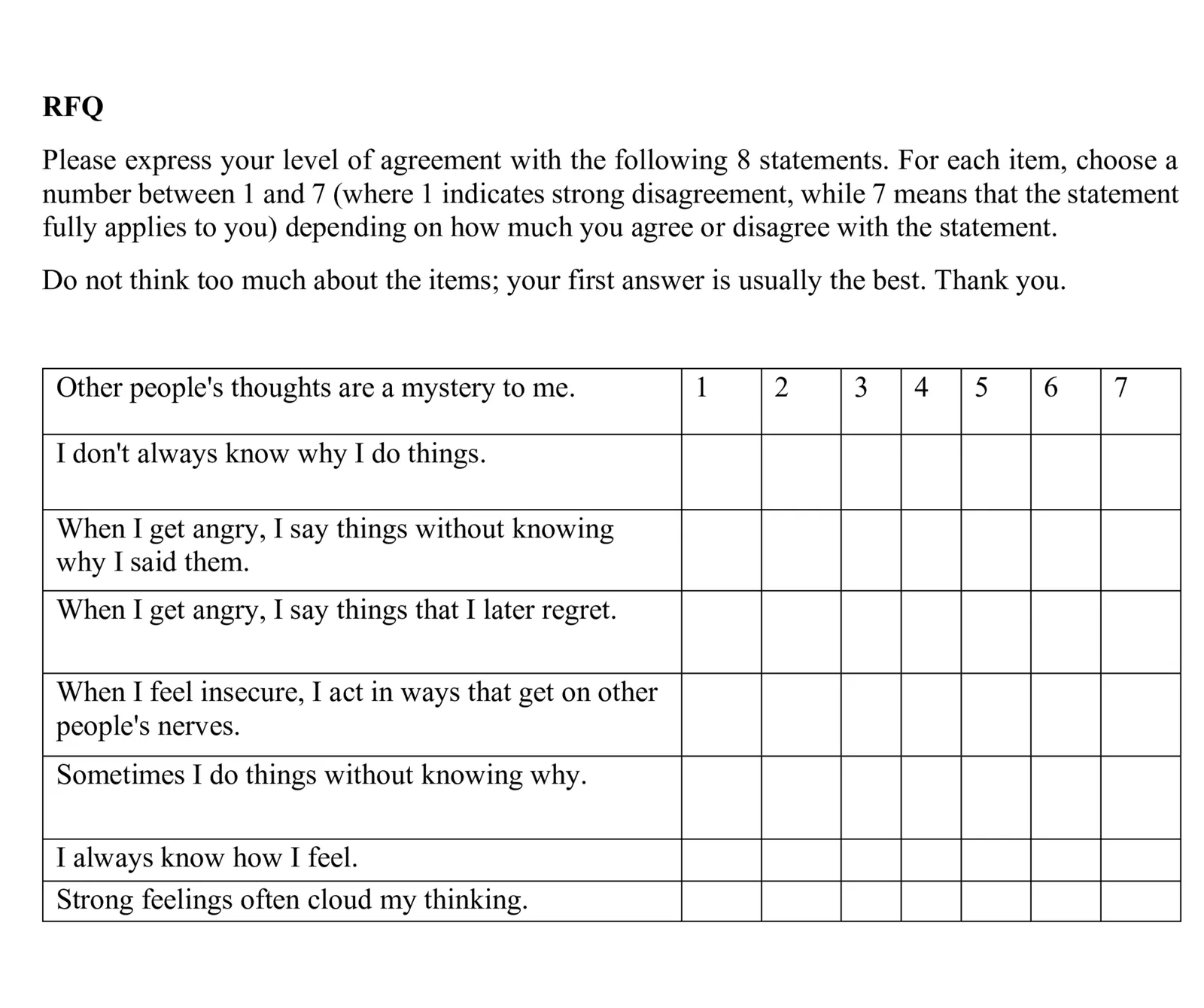
Questionnaire: part 5

## Appendices

Appendix A

Study Questionnaire 
